# On Excision of Joints

**Published:** 1861-09

**Authors:** R. Garner

**Affiliations:** Senior Surgeon to the North Staffordshire Infirmary


### ON EXCISION OF JOINTS.

By R. GARNER, F.L.S., Senior Surgeon to the North Staffordshire Infirmary.

Any information embodying the results of excisions of the large joints is particularly interesting at the present time, especially as regards those of the lower extremity. The merit of the operation on the knee, at least as to its general application, has yet to be decided: it is especially desirable to know in what state the limb is generally found at an advanced period after the operation. With respect to the life of the patient, it has been well ascertained that the operation is tolerably safe; our excisions of the knee at the North Staffordshire Infirmary, five in number, have, apparently, in no instance placed life in danger.

Two adults continue regular patients: the first, a girl of 19, just made out-patient, operated upon on November 24, 1860; the other, a middle-aged man, still an in-patient, in whom the knee was excised on April 6, 1861. Both these cases are considered to be doing very well, both as to the state of the limb and the general health. The young woman still uses crutches, and has some unhealed places on the knee; the man's case is particularly promising, with only one open point. A scrofulous boy having, in addition to the knee, a stiff but quiet elbow, was also operated upon on December 1, 1860, and appears to be going on well, being occasionally brought to the infirmary in a perambulator; the knee firm and straight, but with two open points.*

But with respect to the more important question, the result at a later period of the treatment, the following cases are more interesting: A girl of about seven had her knee excised January 3, 1860, by a U-shaped incision, the patella being left. It may be said that the wound healed by the first intention, and she was discharged well in three months; but upon inquiry (May, 1861) the limb now appears to be a good deal atrophied, the child going about on crutches, and an abscess threatening below the knee. The last case is a young woman of about twenty; excision March, 1860. In April, 1861, the patient used crutches, but could walk a little without them; the leg looked well, but there still existed two unhealed spots in the knee. June 3d.—She can now walk two miles without any support from crutches.

One might at first suppose a person with an excised knee to be in much the same position as a patient with a compound fracture, in which it has been necessary to operate on the ends of the bones; but the probability of recovery with a strong limb is evidently less in the excision, as there is risk of diseased parts being left, with a bad constitution to boot, besides the muscular system being left in an abnormal state in the limb. The treatment of such cases is

---

* August 6, 1861.—The progress of the first patient, the young girl, has not been very favorable, it having been necessary to straighten the limb under chloroform. The leg had been retracted backward, apparently a not uncommon circumstance. It might, perhaps, be well to divide the tendons of the flexor muscle in such cases. The man is still doing well, though it has been necessary to gouge away a prominence of diseased bone. The boy walks without crutch or stick.

very prolonged and expensive, large quantities of wine being commonly consumed, and sometimes removal to the sea-coast being required; but this is of secondary importance to the consideration of the future state of the limb. Without prejudging the case, it may be observed that a weak leg (not reckoning upon its liability to fresh disease) may, in point of utility, be no better than a wooden one; for the laborer the latter would probably be preferable, while the former might be more desired for the counting-house, studio, or drawing-room. The now-ascertained fact that in young subjects the limb often ceases to grow, is a strong argument against the operation in them.

The hip-joint was dextrously excised in one case by Mr. Folker, who also was the operator in two of the above knee cases. It was necessary to gouge the acetabulum, and indeed it was found that the disease could not be totally eradicated. The man sunk of exhaustion ten weeks after the operation, expressing, however, that his sufferings had been alleviated by it.

A friend of the compiler of this paper, Mr. Davis, in his presence very cleverly excised the whole of the ankle in a youth, a private patient, in January, 1858. In May, 1861, the joint had perfectly healed and was sound, but, as is the case in other instances on record, its looseness prevents its being competent to support the body in a very satisfactory manner. In fact, the whole limb is extremely atrophied.

In four adults brought into the infirmary, with compound dislocation or fracture, it has been necessary, or at least thought best, to excise the astragalus. Two of these patients did well, and have now useful limbs; two, however, died of bad diffused inflammation and its disastrous sequelæ.

Our cases of excision of the elbow for disease have been only five, with two partial ones in cases of accident, which did well. Of the five, three were perfectly successful. One, a middle-aged woman, wished for amputation some months after the operation, which was, consequently, done, with recovery. The fifth was the case of a scrofulous and consumptive girl, in whom a portion of the opposite hand had been previously removed; the patient died, I believe, some weeks after the resection, the joint showing no disposition to heal or consolidate. One difficulty in this case was to keep the ends of the bones from riding over each other. It might be well to try the effect of sawing the bones obliquely to prevent the last occurrence, and this might obviate a tendency of the arm to become extended when getting well, which is sometimes a troublesome circumstance. A friend, who has experience in cases of excision, has, in the knee, sawn across the bones, so that, when they were brought into apposition, the leg was a little bent, and in consequence, when cured, practically longer, from the patient walking on the front of the foot; and this he did well.

Mr. Bent, of Newcastle-under-Lyme, afterward surgeon to our infirmary, excised the head of the humerus in 1771, the case being the third excision of any large joint in England. The scapula appears to be often implicated in diseases of the shoulder-joint, at least so we have found it post-mortem; such cases frequently get well without operation.

Certain operations on the long bones may justly be called excisions or resections. In one case the whole of the ulna, except the upper quarter and a bit of the lower extremity, was removed in a young girl. There was no surrounding bony deposit in this case, and the ulna, when loosened at its extremities, slipped out, as it were, into the operator's hand, the arm recovering, with its use unimpaired in all respects. In other cases, portions of diseased fibula have been removed with the best effect, or even of the tibia. In a case of the latter kind, where a good piece of the bone was taken away, the fibula prevented the approach of the extremities of the tibia, and the intervening chasm was not filled up, from the deficiency of bony deposit; in this apparently hopeless state the patient, in desperation, took to walking, and the result was, that the ends of the tibia were brought nearer together, and firm union took place. The leg was, of course, a little bowed, which, however, was of trifling consequence, the patient being a female, and her dress concealing the want of symmetry.—(*Med. Times and Gazette.*)

